# The comparative efficacy and risk of harms of the intravenous and subcutaneous formulations of trastuzumab in patients with HER2-positive breast cancer: a rapid review

**DOI:** 10.1186/s13643-019-1235-x

**Published:** 2019-12-11

**Authors:** Miriam Van den Nest, Anna Glechner, Maria Gold, Gerald Gartlehner

**Affiliations:** 1 Department for Evidence-based Medicine and Clinical Epidemiology, Donau-Universität Krems, Dr.-Karl-Dorrek-Straße 30, 3500 Krems, Austria; 2 Department for Oncology, University Hospital, 3100 St, Pölten, Austria; 3 RTI International, 3040 Cornwallis Rd, Research Triangle Park, Durham, NC 27709 USA

**Keywords:** Breast cancer, Trastuzumab, Route of administration, Rapid review, Subcutaneous

## Abstract

**Background:**

Trastuzumab is a monoclonal antibody for patients with HER2 (human epidermal growth factor receptor 2)-positive breast cancer, which is added to regular treatment and reduces mortality. Originally, trastuzumab had to be administered intravenously (IV) over 30 min every 3 weeks for 1 year. Since 2012, a formulation for the subcutaneous (SC) administration of trastuzumab has been available, which has not yet been approved in the USA. Advocates claim that the SC formulation saves time and money, despite higher costs. The purpose of this study is to review existing literature concerning the comparative efficacy and risk of harms of trastuzumab IV and SC concerning patient-relevant health outcomes.

**Methods:**

We conducted searches in the Cochrane Library and MEDLINE for articles published through May 2018 in English or German. In addition, we searched ClinicalTrials.gov to identify unpublished studies. We dually reviewed the abstracts and full-text articles based on a priori defined inclusion criteria, rated the risk of bias of included studies, and assessed the strength of the evidence for each outcome of interest. Because data was insufficient for quantitative synthesis, we summarized results narratively.

**Results:**

We identified three RCTs (randomized controlled trials) meeting our eligibility criteria, which included data on 1003 patients. We found moderate evidence for similar event rates (20.05% vs. 18%, HR (hazard ratio) 0.88, CI 95% = 0.62–1.27), and mortality rates (10% vs. 8%, HR 0.76, CI 95% = 0.44–1.32) after 1.7 years for patients receiving trastuzumab IV and for patients receiving SC. Results remained similar after 3.3 years, though evidence lacked strength due to a high dropout rate. All trials reported more adverse events among the SC group than in the IV group. Evidence for these findings was of moderate strength. Nevertheless, more than 85% of the patients preferred trastuzumab SC over IV. Results concerning serious adverse events appeared to be heterogeneous.

**Conclusion:**

Results of studies indicate similar efficacy between the two routes of administration. The higher rates of adverse events for SC administration were mainly attributable to injection site–related events. The clinical decision of whether to administer trastuzumab SC or IV requires the consideration of several factors and should be determined individually.

## Background

About 12 to 26% of breast cancer patients are human epidermal growth factor receptor 2 (HER2)-positive [1, 2]. HER2-positive indicates that the tumor cells overexpress HER2-receptors inducing excessive cell proliferation and decreased apoptosis [3]. HER2-positive breast cancers are associated with aggressive biologic behavior and a poorer prognosis than HER2-negative breast cancers [4]. Trastuzumab (trade name, Herceptin®) is a monoclonal antibody that stops the growth of cancer cells by binding to HER2 receptors [3]. Trastuzumab was approved in the USA for the treatment of HER2-positive metastatic breast cancer in 1998 [5]; it is also included on the WHO’s (World Health Organization) List of Essential Medicines [6].

In combination with chemotherapy and/or endocrine therapy, trastuzumab has become standard therapy for women with HER2-positive metastatic breast cancer [7, 8]. Several randomized controlled trials (RCTs) have shown that adding trastuzumab to the treatment regimen improves clinical outcomes for women with HER2-positive breast cancer [9–11]. A Cochrane review, published in 2014, summarized the results of seven RCTs with 1497 patients [12]. Results showed that trastuzumab leads to improved overall survival (HR (hazard ratio) 0.82, 95% CI (confidence interval) = 0.71–0.94) and progression-free survival (HR 0.61, 95% CI = 0.54–0.70) in HER2-positive women with metastatic breast cancer. Side effects of trastuzumab therapy include an increased risk of congestive heart failure (HR 3.49, 95% CI = 1.88–6.47). The initial formulation of trastuzumab requires an intravenous (IV) route of administration starting with a loading dose of 4 to 8 mg/kg over 90 min [13]. If well tolerated, the maintenance dose is 2 mg/kg weekly or 6 mg/kg every 3 weeks over a 30-min period for 52 weeks. A subcutaneous (SC) formulation of trastuzumab has been available since 2012 [14] and was approved by the EMA (European Medicines Agency) in 2013 [15] but has yet to be approved by the FDA (Food and Drug Administration). The SC formulation uses recombinant human hyaluronidase that transiently increases absorption and dispersion of large therapeutic proteins such as monoclonal antibodies after SC application [14]. The time involved in the SC injection of trastuzumab is only 2–5 min [16].

Consequently, trastuzumab SC has the potential to be a time-saving intervention for both patients and health care personnel [17, 18]. The patient “chair time” per cycle (the time from entering the infusion chair until exiting the chair) is, on average, 53.7 min shorter when trastuzumab is administered subcutaneously rather than intravenously. The overall time spent by patients in a hospital is 122.5 min shorter when trastuzumab is administered subcutaneously instead of intravenously (172.7 versus 50.2 min). This totals to about 37 h less over the course of 18 treatment cycles. However, the cost per vial of the SC formulation is approximately three times higher than for the IV formulation (1684 Euros versus 556 Euros) [19]. This means that the total cost difference in medicine alone for an 18-treatment cycle adds up to 20,304 Euros. Nonetheless, several studies, financed by the producer of trastuzumab SC, claimed that the overall costs of using trastuzumab IV (primarily due to increased personnel involvement) were higher than those of trastuzumab SC [19–21]. Several recent randomized controlled trials compared the use of IV and SC formulations of trastuzumab in patients with HER2-positive breast cancer [22–24]. To our knowledge, however, the two different formulations have not been compared systematically regarding patient-relevant health outcomes. The objective of our review was to assess the comparative efficacy and risk of harms of trastuzumab IV and SC formulations in women with HER2-positive metastatic breast cancer.

## Methods

Compared with a systematic review, we employed the following methodological shortcuts in this rapid review: (1) we limited electronic databases to MEDLINE and the Cochrane Library, (2) we limited searches to German and English languages, and (3) we did not register the protocol of the review.

The following sections provide more detail about our methodological approach.

### Literature search

We searched Ovid MEDLINE and the Cochrane Library in May 2018 using *trastuzumab*, *application routes*, and *study designs* as search concepts. When possible, we used both subject headings (MeSH) and free text in our searches. We ran a similar article search for the first 100 linked articles in PubMed using publications [24–26] identified through preliminary searching (see Additional file 1 for complete search strategies). We limited searches to studies on humans published in German and English from inception to the search date. An experienced information specialist conducted these searches. In addition, we searched the register ClinicalTrials.gov and reference lists of included studies and reviews.

### Eligibility criteria

We included studies addressing our predefined inclusion criteria outlined in Table 1.

**Table 1:** Eligibility criteria of included studies

Population	Women with HER2-positive breast cancer
Intervention	Neoadjuvant or adjuvant treatment with subcutaneous trastuzumab
Control	Neoadjuvant or adjuvant treatment with intravenous trastuzumab
Outcomes	• Efficacy (overall survival, event-free survival) • Safety: overall risk of adverse events, serious adverse events, discontinuation because of adverse events • Patients’ preferences
Study design	RCTs, systematic reviews, and meta-analyses

*HER* human epidermal growth factor receptor, *RCT* randomized controlled trial

### Study selection

Two reviewers (A.G. and M.V.) independently screened abstracts and full-text articles using Covidence systematic review software [27]. We developed and pilot-tested abstract and full-text review forms based on the inclusion and exclusion criteria presented above. We retrieved full-text articles of all potentially relevant citations. Reviewers resolved conflicts in decisions regarding inclusion or exclusion by consensus.

### Data abstraction

We abstracted information on population characteristics, interventions, route of administration, and outcomes of interest. One reviewer (M.V.) conducted data abstraction, a second reviewer (A.G.) checked data for completeness and correctness.

### Risk of bias assessment

Both reviewers mentioned above independently assessed the risk of bias of randomized controlled trials using the Cochrane risk of bias tool [28]. They assessed the risk of bias for each outcome of interest and resolved differences by consensus.

### Grading the strength of evidence

We graded the strength of evidence based on the guidance established for the Evidence-based Practice Center Program of the Agency for Healthcare Research and Quality [29]. This approach incorporates four key domains: risk of bias, consistency, directness, and precision of the evidence. It also considers other domains that may be relevant for some scenarios, such as a dose-response association, plausible confounding that would decrease the observed effect, strength of association (magnitude of effect), and publication bias. Grade reflects the strength of the body of evidence to answer questions on the comparative efficacy and risk of harms of subcutaneous versus intravenous application of trastuzumab. Two reviewers (A.G., G.G., and M.V.) independently assessed the strength of evidence for each outcome; they resolved differences by consensus.

### Data synthesis

Because data were insufficient for quantitative analyses, we report findings narratively.

## Results

### Studies

**Fig. 1:**
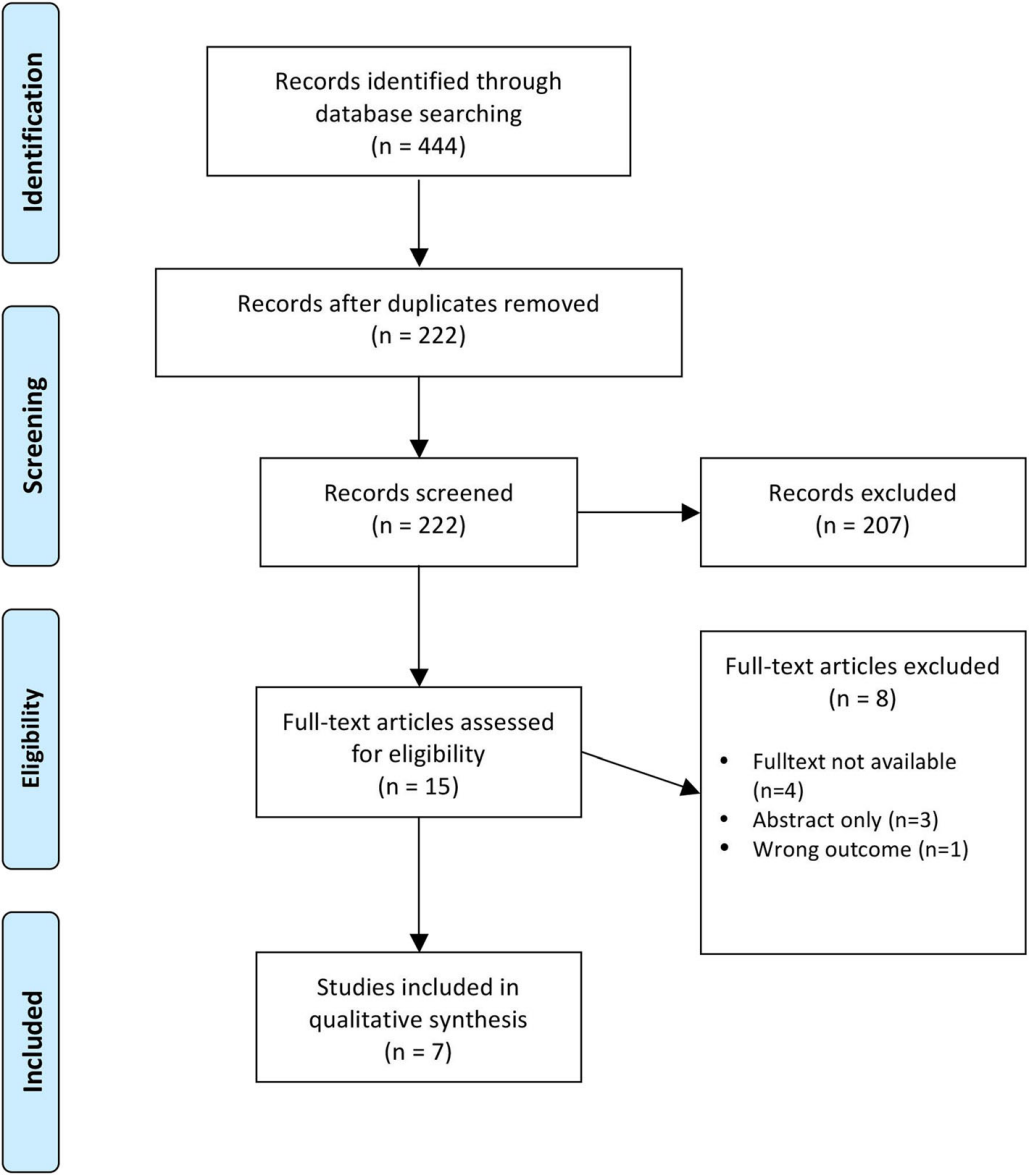
PRISMA flowchart of included studies

Our searches detected 222 citations after deduplication of records of which seven met our eligibility criteria (Fig. 1). After screening titles and abstracts, 207 articles were excluded, as they were considered irrelevant for our questions of interest. Additional eight records were judged unsuitable because full-text publications were not retrievable or studies were published as abstracts only. In one case, the type of comparison (safety after switching between IV and SC applications) was not eligible (Fig. 1). Included publications summarized three RCTs, two of them reporting long-term follow-up data [22–26, 30]. The producer of trastuzumab SC funded all three studies.

The largest study (HannaH (enHANced treatment with NeoAdjuvant Herceptin)), was a parallel-group, open-label RCT (*n* = 595) directly comparing IV with SC administration of trastuzumab during 1 year of treatment and 2 years of treatment-free follow-up [22]. In the HannaH study, more than 90% of women had a tumor stage of T2 or higher [22]. The other two RCTs were crossover trials [24, 26]. In the PrefHer (Preference for Herceptin SC or IV administration) study, 488 women (the majority with T1 or T2 cancers) received four cycles of trastuzumab IV or trastuzumab SC (either via single-injection device or hand-held syringe) and then switched to the other route of administration for another four cycles of treatment [26]. The MetaspHer-study (multicenter, open-label, single-arm safety study of subcutaneous trastuzumab in combination with pertuzumab and docetaxel in patients with HER2-positive advanced breast cancer) enrolled 113 women with metastasizing breast cancer [24]. After three cycles of trastuzumab (IV or SC), women crossed over to the other route of administration for another three cycles of treatment. Table 2 summarizes study and population characteristics.

**Table 2:** Baseline characteristics of included studies

	HannaH 2015		PrefHer 2017	MetaspHer 2017
	Trastuzumab SC (*n* = 294)	Trastuzumab IV (*n* = 297)	*n* = 483	*n* = 113
Design	RCT		RCT, crossover	RCT, crossover
Follow-up	52–172 weeks		24 weeks	18 weeks
Age (years)	50 (25–81)	50 (24–77)	53 (27–83)	59 (35–85)
Body weight (kg)	68 (39–126)	66 (42–137)	66 (41–117)	70 (46–110)
Breast cancer subtype				
Ductal	272 (92.8%)	273 (91.9%)	Not specified	70 (87.5%)
Lobular	12 (4.1%)	17 (5.7%)		3 (3.8%)
Other	9 (3.1%)	7 (2.4%)		6 (7.5%)
Ductal + other	0	0		1 (1.3%)
Estrogen receptor status				
Negative	140 (47.6%)	148 (49.8%)	168 (34.8%)	43 (38.1%)
Positive	154 (52.4%)	148 (49.8%)	309 (64%)	57 (50.4%)
Unknown	0	1 (0.3%)	6 (1.2%)	13 (11.5%)
Nodal status				
Negative	71^a, b^ (24.2%)	62^a^ (20.9 %)	229 (49.04%)^c^	Not specified
Positive	222^a, b^ (75.8%)	235^a^ (88 %)	227 (48.61%)^c^	
Unknown	0	0	11 (2.36%)^c^	
Tumor stage				
T0	0	0	5 (1%)	Not specified
T1	19 (6.5%)^d^	23 (7.9%)^d^	204 (42.2%)	
T2	129 (44%)^d^	130 (43.8%)^d^	208 (43.1%)	
T3	52 (17.7%)^d^	49 (16.5%)^d^	37 (7.7%)	
T4	93 (31.7%)^d^	95 (32%)^d^	25 (5.2%)	
Unknown	0	0	4 (0.8%)	

^a^Clinical nodal status, ^b^*n* = 293, ^c^*n* = 467, ^d^clinical tumor stage

In total, the three RCTs provide data on 1187 women with HER2-positive breast cancer. Follow-up was 18 weeks to 3.3 years. The median age of women ranged from 50 to 60 years (Table 2) [22–24]. No specific restrictions for age or ethnicity were stated in any of the RCTs, for the HannaH trial most patients were of white ethnicity (68.4%), followed by Asian (21.2%), and other (10.5%) ethnicities. Most cancers were of a ductal subtype, with a slight majority being estrogen-receptor-positive. All three trials determined the comparative risks of IV and SC administration. Only one trial reported efficacy data [22]. We rated the included studies as low and high risk of bias for efficacy outcomes (depending on time of follow-up and respective drop-out rates) and unclear or high risk of bias for the assessment of adverse events (Table 3).

**Fig. 2:**
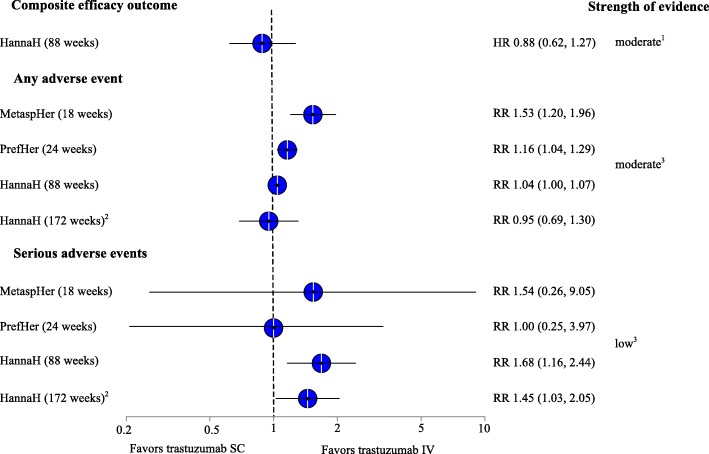
Summary of results for efficacy and safety outcomes*. Abbreviations*: *HR* hazard ratio, *IV* intravenous, *RR* risk ratio, *SC* subcutaneous. ^1^Due to imprecision, ^2^drop-out rate of 37%, ^3^due to high risk of bias, ^4^due to high risk of bias and imprecision

Figure 2 summarizes efficacy and adverse events results of included studies graphically.

The following sections provide more detail about effects in individual studies. Additional file 2 presents the strength of evidence for individual outcomes.

### Efficacy

Only the HannaH study reported data comparing the efficacy of trastuzumab IV (8 mg/kg loading dose, 6 mg/kg maintenance dose) and trastuzumab SC (600 mg) [25, 30]. The primary endpoints were the serum trough concentration of trastuzumab prior to surgery, and the pathological complete response (defined as the absence of neoplastic invasive cells), which did not meet our inclusion criteria because of the intermediate nature of such outcomes. As a secondary efficacy outcome, however, the HannaH study assessed a composite measure of relapse, progression of disease, or death after 1.7 and 3.3 years of follow-up.

Based on this health outcome, results indicate similar efficacy between the two routes of administration. After 1.7 years, 20.5% (61 out of 297) of women in the group with IV administration had experienced an event of the composite outcome (relapse, progression of disease, or death), compared with 18.0% (53 out of 294) in the SC administration group (HR 0.88, 95% CI = 0.62–1.27) (Additional file 2) [25]. After 3.3 years, composite event rates increased to 26.9% (80 out of 297) and 24.1% (71 out of 294), respectively. The difference between the groups remained statistically non-significant (HR 0.95, 95% CI = 0.69–1.30) (Table 3) [30]. Likewise, mortality rates were similar between women treated with trastuzumab IV (10%; 30 out of 297) or trastuzumab SC (8%, 24 out of 294; HR 0.76, 95% CI 0.44–1.32). We rated the strength of evidence as moderate that the efficacy of trastuzumab IV and SC is similar regarding health outcomes after 1.7 years and as low after 3.3 years of follow-up.

### Safety

In all three included studies, women randomized to SC injections reported higher rates of adverse events than women receiving IV administration of trastuzumab [22, 24, 26]. The higher rates of adverse events for SC administration were mainly attributable to injection site–related events, such as pain or erythema. In one of the crossover trials (PrefHer), 62.6% (300 out of 479) of women reported adverse events during the SC administration period, compared with 54% (258 out of 478) during the IV period (RR (risk ratio) 1.16, 95% CI 1.04–1.29) (Additional file 2) [26]. The difference in overall risks of adverse events was caused primarily by injection-related reactions, such as pain (6.7%) or erythema (5.8%), which appeared only during the SC administration. Other adverse events were similar between the IV and SC periods, including asthenia (5.2% vs. 6.3%), arthralgia (5.6% vs. 5.2%), hot flushes (3.6% vs. 4.6%), fatigue (3.8% vs. 4%), headache (3.6% vs. 4.2%), and diarrhea (2.5% vs. 3.3%). The HannaH and MetaspHer-study reported similar findings (Fig. 2).

In the HannaH study, statistically significantly more patients in the trastuzumab SC than in the trastuzumab IV group experienced serious adverse events (12.4% vs. 20.9%; RR 1.68, 95% CI 1.16–2.44) and discontinued due to adverse events (2.3% vs. 5.7%; RR 2.44, 95% CI 1.03–5.79) [22]. The difference in serious adverse events was caused mainly by serious general infections and infestations, which occurred in 4.3% vs. 8.8% of the patients [31], respectively (RR = 1.85, 95% CI = 0.96–3.57). Serious infections included infections of the respiratory tract (1.4% vs. 3.4%), cellulitis (0% vs. 0.7%), or post-operative wound infections (0% vs. 0.7%), though most of them were single cases and seemed not to be related to treatment with trastuzumab, except for one case of post-procedural infection, which was not further described. Other serious adverse events, like febrile (3.4% vs. 4.4%) and other neutropenia (3% vs. 2.4%), cardiac adverse events (1% vs. 2%), and pulmonary and respiratory adverse events (0.7% vs. 1.3%) were rare. There was one case of decreased ejection fraction in the SC group (0.3%) and two cases of hypersensitivity in the IV group (0.7%).

The PrefHer- and the MetaspHer-study reported only few serious adverse events in each group (0.8% vs. 0.8% and 1.8% vs. 2.9%) [24, 26]. In PrefHer, four patients in each group experienced serious adverse events unrelated to treatment with trastuzumab. After the crossover periods, one patient who continued on trastuzumab SC, suffered from serious left ventricular dysfunction. Further, the rate of withdrawal due to adverse events in this study was similar between both groups (1.3% vs. 1%). The MetaspHer-trial did not report on discontinuation on account of side effects [24]. Overall, due to inconsistent results across studies, imprecision, and serious risk of bias (lack of blinding) the evidence is insufficient to draw inferences about differences in risks for serious adverse events.

### Patient preferences

Both crossover RCTs examined the preferences of women concerning the two different formulations of trastuzumab. In both studies, women preferred SC over IV administration of trastuzumab (88.9% and 85.9%) [23, 24]. This preference seemed to be due to time saved and, contrary to the rates of adverse events reported by clinicians, to less pain or discomfort [23].

## Discussion

The aim of this study was to review the literature examining the comparative efficacy and safety of subcutaneous trastuzumab with the established intravenous formulation. Our searches yielded only one trial for the comparative efficacy [22, 25, 30] and three studies comparing the safety of trastuzumab IV and SC [24–26]. This body of evidence suggests similar efficacy but a slightly increased risk of adverse events for trastuzumab SC. Efficacy results, however, are limited by a high dropout rate after the 3-year mark [30]. Thus, the strength of evidence is low regarding similar efficacy of trastuzumab IV and SC. All three studies reported more adverse events during SC than IV administration, though most of these events seemed to be mild [22–26]. In the largest trial, which administered trastuzumab in addition to chemotherapy, numerically more patients in the SC group experienced serious adverse events than in the IV group [22]. This difference, however, did not reach statistical significance. Despite the higher rate of side effects reported by clinicians, most patients preferred trastuzumab SC over IV, mainly due to time saved and less discomfort or pain [23, 24]. For all of the trials, masking of outcome assessors and patients was either not conducted or unclear.

Our rapid review has several limitations, some of which are inherent to rapid reviews and abbreviated methods. First, we searched only two databases (MEDLINE and Cochrane which includes the Cochrane Central Register of Controlled Trials (CENTRAL)) for articles restricted to German or English. CENTRAL is the largest database of RCTs but it does not cover the most recent publications with a time lag of about 6 months. Consequently, there is a small risk that we may have missed very recent publications in languages other than German or English. Second, some of our conclusions are uncertain because of sparse and heterogeneous data. The lack of certainty is reflected in low strength of evidence ratings, which indicates that future studies might have a substantial impact on effect estimates. Third, all included studies were funded by Hoffmann-La Roche, the producer of trastuzumab SC. Methods research has shown that funding bias can lead to findings that favor the intervention associated with the funding source [32]. Finally, publication bias remains a threat for all evidence syntheses. We have no way to tell whether all studies comparing trastuzumab IV and SC actually have been published. Searches in ClinicalTrials.gov did not detect any registered trials that have been finished but remained unpublished. We further did not enroll our study protocol at a trial register, despite their importance in reducing publication bias and selected reporting.

We found three ongoing crossover trials examining the preferences and safety concerning trastuzumab IV and SC [33–35], which are soon to be finished. Another RCT with 500 patients that compares the efficacy and safety of the two formulas was initiated in 2018 [36].

The clinical implications of our findings have to be interpreted within the context of clinical practice. A survey of physicians of seven German tertiary and university hospitals revealed that trastuzumab SC was the preferred option if no other drug therapy was administered in parallel [37]. Patients with a central venous port, who were receiving chemotherapy or another antibody therapy, received intravenous trastuzumab to avoid additional injections. Avoiding additional injections seems preferable although time could be saved by using the subcutaneous administration route. If, in the future, trastuzumab SC can be administered by a local general practitioner or a nurse at home, additional hospital administration costs would be minimized or eliminated.

In conclusion, the two different administration routes had comparable efficacy with a higher rate of local adverse events with trastuzumab SC. Given similar efficacy and safety, the clinical decision regarding the administration route of trastuzumab needs to have a strong focus on patient preferences and cost consideration.

## Supplementary information

**Additional file 1.** Complete search strategy, this file contains the complete search strategy for our literature search in MEDLINE, CENTRAL and Pubmed

**Additional file 2.** Strength of evidence for main outcomes, this file reports the strength of evidence for safety and efficacy outcomes of all the trials

## Abbreviations

CENTRAL: Cochrane Central Register of Controlled Trials
CI: Confidence interval
FDA: Food and Drug Administration
HER-2: Human epidermal growth factor 2
HR: Hazard ratio
IV: Intravenous
RCT: Randomized controlled trial
RR: Risk ratio
SC: Subcutaneous
WHO: World Health Organization
