# Supplementary material for: The comparative efficacy and risk of harms of the intravenous and subcutaneous formulations of trastuzumab in patients with HER2-positive breast cancer: a rapid review
Source: Syst Rev. 2019 Dec 11;8:321. doi: 10.1186/s13643-019-1235-x (PMC6905114; doi:10.1186/s13643-019-1235-x)
Supplement: Supplementary file 1 — Additional file 1. Complete search strategy, this file contains the complete search strategy for our literature search in MEDLINE, CENTRAL and Pubmed [file 13643_2019_1235_MOESM1_ESM.pdf]

Additional File 1. Complete search strategy

Ovid Medline

08. Mai 2018

Ovid MEDLINE(R) 1946 to April Week 4 2018, Ovid MEDLINE(R) Epub Ahead of Print May 08, 2018, Ovid MEDLINE(R) In-Process & Other Non-Indexed Citations May 07, 2018, Ovid MEDLINE(R) Daily Update May 08, 2018

| # | Searches | Results |
|---|---|---|
| 1 | antibodies, monoclonal, humanized/ or trastuzumab/ | 32960 |
| 2 | (trastuzumab or ogivri or herceptin).ti,ab. | 8748 |
| 3 | (anti* adj3 (HER2 or ERB-2)).ti,ab. | 2774 |
| 4 | 1 or 2 or 3 | 38101 |
| 5 | exp Administration, Intravenous/ | 136309 |
| 6 | intravenous*.ti,ab. | 314819 |
| 7 | (iv adj3 (infus* or inject* or admin* or route* or treatment* or formula*)).ti,ab. | 50844 |
| 8 | 5 or 6 or 7 | 408143 |
| 9 | infusions, subcutaneous/ or exp injections, subcutaneous/ | 39177 |
| 10 | subcutaneous*.ti,ab. | 150155 |
| 11 | (sc adj3 (infus* or inject* or admin* or route* or treatment* or formula*)).ti,ab. | 14326 |
| 12 | 9 or 10 or 11 | 181465 |
| 13 | 4 and 8 and 12 | 327 |
| 14 | exp animals/ not exp humans/ | 4453926 |
| 15 | 13 not 14 | 314 |
| 16 | (english or german).lg. | 24643129 |
| 17 | 15 and 16 | 309 |
| 18 | (systematic or structured or evidence or trials).ti. and ((review or overview or look or examination or update* or summary).ti. or review.pt.) | 129332 |
| 19 | (0266-4623 or 1469-493X or 1366-5278 or 1530-440X).is. | 15742 |
| 20 | meta-analysis.pt. or Network Meta-Analysis/ or (meta-analys* or meta analys* or metaanalys* or meta synth* or meta-synth* or metasynth*).tw,hw. | 154025 |
| 21 | review.pt. and ((medline or medlars or embase or pubmed or scisearch or psychinfo or psycinfo or psychlit or psyclit or cinahl or electronic database* or bibliographic database* or computeri#ed database* or online database* or pooling or pooled or mantel haenszel or peto or dersimonian or der simonian or fixed effect or ((hand adj2 search*) or (manual* adj2 search*))).tw,hw. or (retraction of publication or retracted publication).pt.) | 128464 |
| 22 | ((systematic or meta) adj2 (analys* or review)).ti,kf. or ((systematic* or quantitativ* or methodologic*) adj5 (review* | 187878 |

| | | |
|---|---|---|
| | or overview*)).tw,hw. or (quantitativ$ adj5 synthesis$).tw,hw. | |
| 23 | (integrative research review* or research integration).tw. or scoping review?.ti,kf. or (review.ti,kf,pt. and (trials as topic or studies as topic).hw.) or (evidence adj3 review*).ti,ab,kf. | 170476 |
| 24 | 18 or 19 or 20 or 21 or 22 or 23 | 430702 |
| 25 | 24 not (case report/ or letter.pt.) | 419715 |
| 26 | 17 and 25 | 33 |
| 27 | randomized controlled trial.pt. or (random* and control* and trial).ti,ab,kf,hw. | 548700 |
| 28 | 17 and 27 | 99 |
| 29 | 26 or 28 | 125 |

Cochrane Library

14. Mai 2018

| ID | Search | Hits |
|---|---|---|
| #1 | [mh ^"antibodies, monoclonal, humanized"] or [mh trastuzumab] | 3286 |
| #2 | (trastuzumab or ogivri or herceptin):ti,ab,kw | 1634 |
| #3 | (anti* near/3 (HER2 or ERB-2)):ti,ab,kw | 267 |
| #4 | {or #1-#3} | 4631 |
| #5 | [mh "Administration, Intravenous"] | 17912 |
| #6 | intravenous*:ti,ab,kw | 71319 |
| #7 | (iv near/3 (infus* or inject* or admin* or route* or treatment* or formula*)):ti,ab,kw | 20464 |
| #8 | {or #5-#7} | 72782 |
| #9 | [mh ^"infusions, subcutaneous"] or [mh "injections, subcutaneous"] | 4305 |
| #10 | subcutaneous*:ti,ab,kw | 19251 |
| #11 | (sc near/3 (infus* or inject* or admin* or route* or treatment* or formula*)):ti,ab,kw | 5473 |
| #12 | {or #9-#11} | 20302 |
| #13 | #4 and #8 and #12 | 139 |
| #14 | #13 in Cochrane Reviews (Reviews and Protocols), Other Reviews, Technology Assessments and Economic Evaluations | 8 |
| #15 | #13 in Trials | 131 |

Pubmed similar articles

14. Mai 2018

| Search | Query | Items found |
|---|---|---|
| #1 | Search 28963915 25403587 28648618[uid] | 3 |
| #2 | Similar articles for PubMed (Search 28963915 25403587 28648618[uid]) | 199 |

| | | |
|---|---|---|
| #3 | Search 28648618 28963915 25403587 28549309 22884505<br>28625777 27208905 25070545 23965225 28215665<br>23764181 25130998 29146401 26181252 24657003<br>24095300 28923573 26806010 22153890 26539793<br>26645407 26822398 25456368 24664187 28950146<br>24074778 26092818 27622751 21354370 28624696<br>27059339 20113825 28335887 23871490 24239210<br>23602601 28733983 28289045 23436264 24051172<br>25524798 22257673 26953588 23234763 27357813<br>26334099 25205424 25640059 26596672 26829011<br>29037983 23382472 21172893 25713436 27167986<br>25623753 24677057 27276706 22094934 21788566<br>28155054 26598744 24080156 27693116 21887681<br>20658263 21729666 27512838 28279941 23328596<br>25935793 16236737 27955684 27379947 25779558<br>28526538 23613387 28238593 24793816 26267412<br>26630533 27100299 29448072 24934637 27271767<br>27306421 23569311 25584488 17208639 19856052<br>27259714 25579459 23479422 20308670 21843921<br>23022045 23957715 28682681 25760024 23613383<br>22377126 29175149 24919460 23562929 20564392<br>22513938 23749924 22772380 17647266 26563257<br>16249641 22648179 28174294 20728210 20709813<br>26389758 18667396 19156515 19331197 25752727<br>21868556 12401903 18490651 15800309 24347519<br>24608202 23878115 14679114 25065563 15582895<br>25913287 24521993 27866859 28526536 27009140<br>27181019 25761487 26975189 26691837 27498127<br>25885598 26166837 28343975 26099745 26834058<br>11821453 25467016 28341959 29373094 28000658<br>27918780 25024001 28056202 19786670 26625004<br>25026897 26099741 28737130 26456898 28399902<br>24903272 17516068 26917603 27041112 25296592<br>27078022 25693012 20124187 25370464 25019376<br>24401928 27107569 29544445 29136405 29511355<br>27111595 27875517 26977267 29330636 26702392<br>29502805 29350568 27425556 29245056 28534353<br>29330204 29113393 28721185 25625444 27284260<br>28577152 28592381 25076790 29199854 27536148<br>26546943 28983334 28252362 27115663[uid] | 199 |
| #4 | Search "Animals"[Mesh] NOT "Humans"[Mesh] | 4450964 |
| #5 | Search (#3 NOT #4) | 199 |
| #6 | Search "english"[Language] OR "german"[Language] | 24606577 |
| #7 | Search (#5 AND #6) | 197 |
| #8 | Search (#7 AND systematic[sb]) | 8 |
| #9 | Search (#7 AND "therapy/narrow"[Filter]) | 71 |
| #10 | Search (#8 OR #9) | 78 |
