# Supplementary material for: The comparative efficacy and risk of harms of the intravenous and subcutaneous formulations of trastuzumab in patients with HER2-positive breast cancer: a rapid review
Source: Syst Rev. 2019 Dec 11;8:321. doi: 10.1186/s13643-019-1235-x (PMC6905114; doi:10.1186/s13643-019-1235-x)
Supplement: Supplementary file 2 — Additional file 2. Strength of evidence for main outcomes, this file reports the strength of evidence for safety and efficacy outcomes of all the trials [file 13643_2019_1235_MOESM2_ESM.docx]

Additional file 2
Strength of evidence for main outcomes

| Studies | | Risk of bias | Inconsistency | | Indirectness | | | Imprecision | Other considerations | Effect (CI95%) | Strength of evidence |
| --- | --- | --- | --- | --- | --- | --- | --- | --- | --- | --- | --- |
| Composite event-rate (relapse, progression of disease, or death) after 1.7 years | | | | | | | | | | | |
| HannaH [25] | 1 RCT | low | | not applicable | | no serious indirectness | serious imprecision^a^ | | none | HR 0.88  (0.62-1.27) | moderate |
| Composite event-rate (relapse, progression of disease, or death) after 3.3 years | | | | | | | | | | | |
| HannaH [30] | 1 RCT | high^b^ | | not applicable | | no serious indirectness | serious imprecision^a^ | | none | HR 0.95  (0.69-1.30) | low |
| **Mortality-rate after 3.3 years** | | | | | | | | | | | |
| HannaH [30] | 1 RCT | high^b^ | | not applicable | no serious indirectness | | serious imprecision^a^ | | none | HR 0.76  (0.44-1.32) | low |
| **Adverse events** | | | | | | | | | | | |
| HannaH [22] | 3 RCTs | high^c^ | | serious inconsistency | | no serious indirectness | no serious imprecision | | none | RR 1.04^1^ (1.00-1.07) | moderate |
| PrefHer [26] |  |  |  |  |  |  |  |  |  | RR 1.16^1^ (1.04-1.29) |  |
| MetaspHer [24] |  |  |  |  |  |  |  |  |  | RR 1.53^1^ (1.20-1.96) |  |

*Abbreviations: CI* Confidential Interval*, HR* Hazard Ratio*, RCT* Randomized Controlled Trial*, RR* Risk Ratio
^1^Self-calculated by the authors
^a^few patients;  ^b^37% dropout-rate; ^c^Lack of masking or unclear masking of patients and outcome assessors, lack of ITT analysis; ^d^few events

Additional file 2
Strength of evidence for main outcomes

| Studies | | Risk of bias | | | Inconsistency | Indirectness | Imprecision | Other considerations | Effect (CI95%) | | Strength of evidence |
| --- | --- | --- | --- | --- | --- | --- | --- | --- | --- | --- | --- |
| **Serious adverse events** | | | | | | | | | | | |
| HannaH [22] | 3 RCTs | | high^c^ | serious inconsistency | | no serious indirectness | serious imprecision^d^ | none | RR 1.68^1^  (1.16–2.44) | insufficient | |
| PrefHer [26] |  |  |  |  |  |  |  |  | RR 1.00^1^  (0.06–15.91) |  |  |
| MetaspHer [24] |  |  |  |  |  |  |  |  | RR 1.54^1^  (0.26–9.05) |  |  |
| Discontinuation due to adverse events | | | | | | | | | | | |
| HannaH [25] | 2 RCTs | | high^c^ | serious inconsistency | | no serious indirectness | serious imprecision^d^ | none | RR 2.44^1^ (1.03-5.79) | insufficient | |
| PrefHer [23] |  |  |  |  |  |  |  |  | RR 0.83^1^  (0.26-2.71) |  |  |

*Abbreviations: CI* Confidential Interval*, HR* Hazard Ratio*, RCT* Randomized Controlled Trial*, RR* Risk Ratio
^1^Self-calculated by the authors
^a^few patients; ^b^37% dropout-rate; ^c^Lack of masking or unclear masking of patients and outcome assessors, lack of ITT analysis; ^d^few events
